# Comparison of tuning properties of gamma and high-gamma power in local field potential (LFP) versus electrocorticogram (ECoG) in visual cortex

**DOI:** 10.1038/s41598-020-61961-9

**Published:** 2020-03-25

**Authors:** Agrita Dubey, Supratim Ray

**Affiliations:** 1 Centre for Neuroscience, Indian Institute of Science, Bangalore, 560012 India; 2 Center for Neural Science, New York University, New York, 10003 USA

**Keywords:** Neural circuits, Striate cortex

## Abstract

Electrocorticogram (ECoG), obtained from macroelectrodes placed on the cortex, is typically used in drug-resistant epilepsy patients, and is increasingly being used to study cognition in humans. These studies often use power in gamma (30–70 Hz) or high-gamma (>80 Hz) ranges to make inferences about neural processing. However, while the stimulus tuning properties of gamma/high-gamma power have been well characterized in local field potential (LFP; obtained from microelectrodes), analogous characterization has not been done for ECoG. Using a hybrid array containing both micro and ECoG electrodes implanted in the primary visual cortex of two female macaques (for some stimulus conditions, separate ECoG and microelectrode arrays in two additional male macaques were also used), we compared the stimulus tuning preferences of gamma/high-gamma power in LFP versus ECoG in up to four monkeys, and found them to be surprisingly similar. High-gamma power, thought to index the average firing rate around the electrode, was highest for the smallest stimulus (0.3° radius), and decreased with increasing size in both LFP and ECoG, suggesting local origins of both signals. Further, gamma oscillations were similarly tuned in LFP and ECoG to stimulus orientation, contrast and spatial frequency. This tuning was significantly weaker in electroencephalogram (EEG), suggesting that ECoG is more like LFP than EEG. Overall, our results validate the use of ECoG in clinical and basic cognitive research.

## Introduction

Electrocorticography (ECoG), also known as intracranial electroencephalography (iEEG), is obtained from macroelectrodes placed subdurally on the pial surface of cortex and is widely used in drug-resistant epilepsy patients. The patients are often monitored for weeks for localization of the seizure focus, allowing (with patient’s consent) researchers to conduct cognitive and neuroscience studies^1–9^.

These studies often use power in gamma (30–70 Hz) and high-gamma (>80 Hz) ranges to make inferences about the underlying neural processing^10^. High-gamma activity (>80 Hz) refers to power over a broad range of frequencies above the gamma band that, in ECoG, is modulated by stimulus presentation as well as the behavioral state^4,5,10–13^. High-gamma activity is also observed in local field potential (LFP) obtained by inserting microelectrodes in the cortex of animals, where it is tightly correlated with the spiking activity of neurons in the vicinity of the microelectrode^13–17^.

Gamma rhythm (30–70 Hz), which is different from high-gamma activity^17^, has been extensively studied in electroencephalogram (EEG) in humans and LFP in animals, and has been associated with high level cognitive functions such as attention, memory and perception^18–24^. Further, gamma is known to be strongly induced by stimuli such as bars/gratings and depends on stimulus properties such as size, orientation, spatial frequency, contrast and temporal frequency^16,17,25–29^. Stimulus dependence of gamma has also been characterized in EEG/MEG studies^30–34^. However, only a few studies have characterized the stimulus preference of gamma in ECoG^35,36^. No study, to our knowledge, has done a direct comparison of stimulus preferences of gamma/high-gamma in LFP versus ECoG.

Apart from providing clues about the neural correlates of gamma/high-gamma activity in ECoG, such a comparison allows us to determine the spatial spread (the cortical area around the electrode that contributes to the signal that is recorded from that electrode) of ECoG, which we have recently shown to be very local^37^. For example, both the firing rates and LFP high-gamma power reduce with increasing stimulus size because of larger surround suppression^17^. However, since a larger stimulus activates a larger cortical area, we might observe an increase in ECoG high-gamma (despite a reduction in firing rate) if ECoG spatial spread is much larger than LFP. Similarly, we have recently shown that gamma power recorded using EEG has much weaker tuning preferences (for stimulus orientation, size and contrast) compared to LFP^29^. A comparison of analogous gamma tuning preferences for ECoG versus LFP will provide clues about their similarity. Recording from a unique hybrid grid which consists of both micro and macro-electrodes, implanted in the primary visual cortex of the same two female macaques for which we had earlier compared LFP versus EEG tuning^29^ and LFP versus ECoG spatial spreads^37^, we compared the strength of ECoG and LFP gamma/high-gamma power for different stimulus properties such as size, orientation, spatial frequency and contrast.

## Results

**Figure 1:**
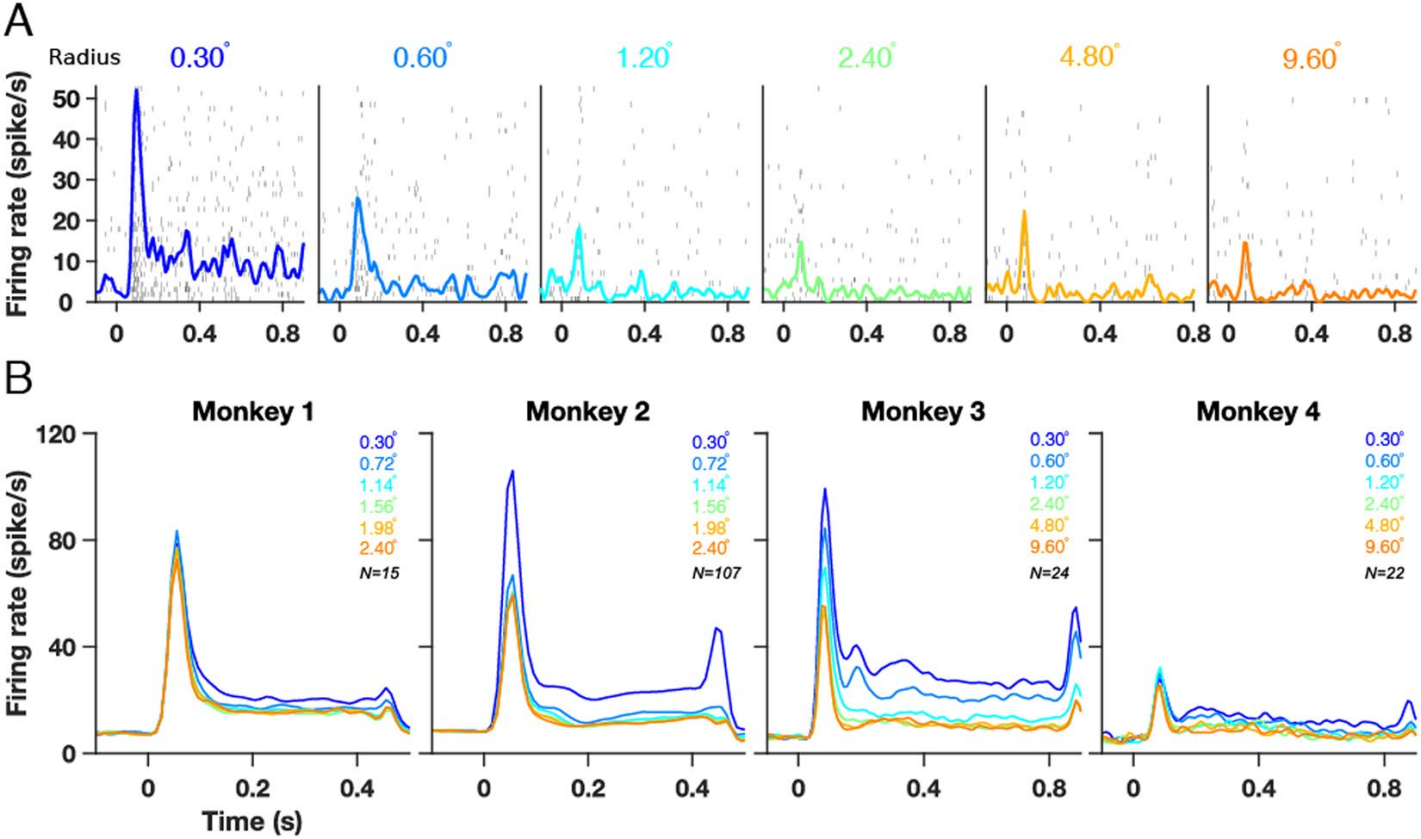
Spiking activity for different stimulus sizes. (**A**) Raster plots showing spiking activity in individual trials for each stimulus size for an example unit from Monkey 3. Each row represents a trial. The peristimulus histogram, averaged across trials is overlaid on the raster plots. **(B)** Averaged firing rates for six stimulus sizes shown as different color traces for Monkeys 1, 2, 3 and 4.

**Figure 2:**
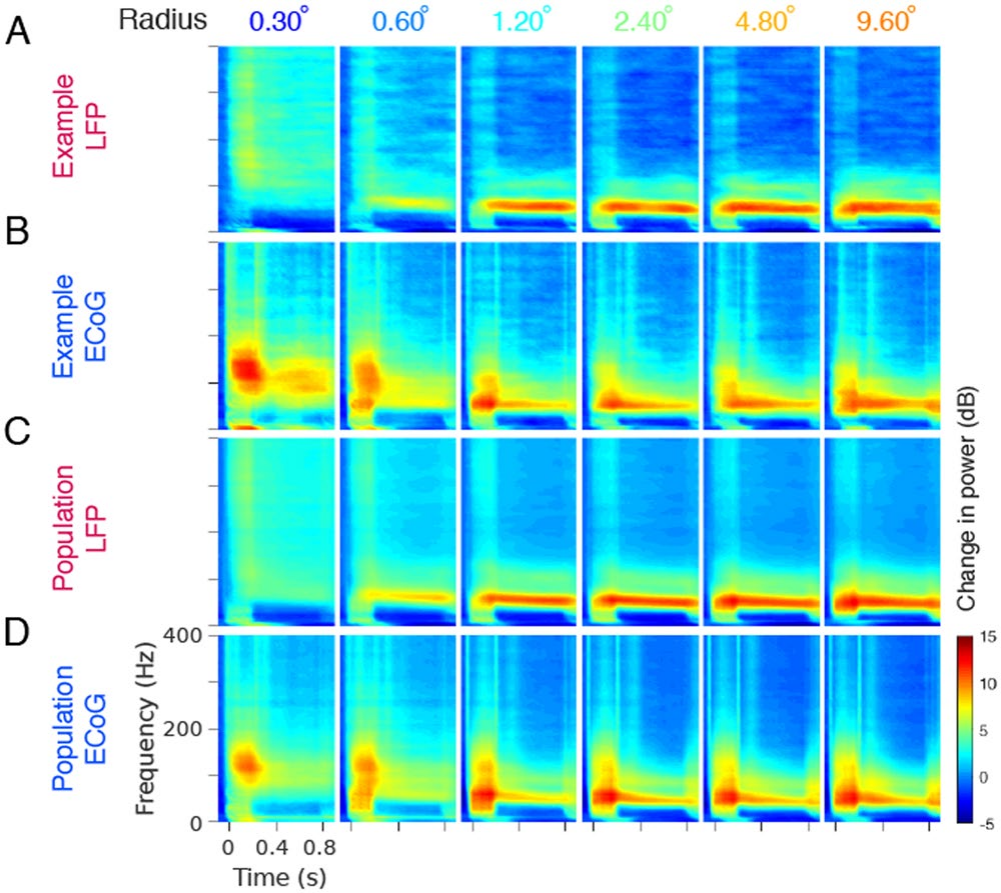
Gamma oscillations and high-gamma activity as a function of stimulus size in LFP and ECoG for Monkey 3. (**A**) Time-frequency energy difference plots (in dB) showing the difference in energy relative to baseline energy (−500 to 0 ms, 0 denotes the stimulus onset, stimulus is presented from 0 to 800 ms) for six stimulus radii (labelled above the plots in degrees) for an example LFP recording site (same as shown in Fig. 1A). The gamma rhythm at ~50 Hz increases with size, while the high-band activity above the gamma band decreases with size. **(B)** same as A for an example ECoG recording site. **(C,D)** show the corresponding population responses of 24 LFP and 5 ECoG recording sites.

**Figure 3:**
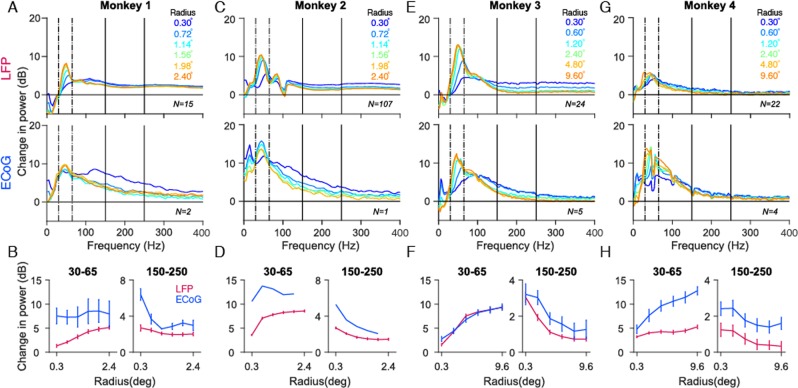
Tuning of gamma oscillations and high-gamma activity for stimulus size. **(A**,**C)** Average relative change in power spectra between 200 and 400 ms from baseline energy (−200 to 0 ms) for 15 and 107 LFP recordings sites (top panel), 2 and 1 ECoG recording sites (bottom panel) for Monkeys 1 and 2. **(E,G)** same as A, C but for 24 and 22 LFP recordings sites (top panel), 5 and 4 ECoG recording sites (bottom panel) for Monkeys 3 and 4. The change in power is computed between 250 to 750 ms relative to baseline energy (−500 to 0 ms). **(B,D,F,H)** Change in LFP (magenta) and ECoG (blue) for gamma (30–65 Hz) and high-gamma (150–250 Hz) frequency bands as a function of stimulus size. Error bar indicates SEs of the mean. Note that the stimulus radii for Monkeys 1 and 2 are different from Monkeys 3 and 4.

**Figure 4:**
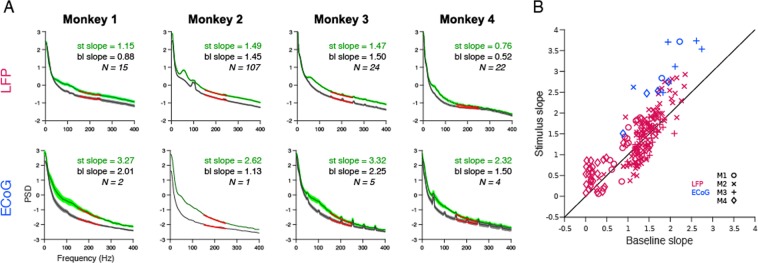
Slope of the high-gamma activity for 0.3° stimulus. (**A**) Average LFP (top panel) and ECoG (bottom panel) for stimulus period (green traces, 200 to 400 ms for Monkeys 1 and 2; 250 to 750 ms for Monkeys 3 and 4) and baseline period (black trace, −200 to 0 ms for Monkeys 1 and 2; −500 to 0 ms for Monkeys 3 and 4, where 0 ms is the stimulus onset). Standard errors of the mean is shown in a lighter shade. **(B)** The slope of LFP (magenta) and ECoG (blue) electrodes computed for high-gamma frequency range (150–250 Hz) for baseline period is plotted in x-axis and for stimulus period in y-axis. The four monkeys are represented using four different marker types.

We simultaneously recorded LFP and ECoG signals using a special custom-made hybrid grid electrode array implanted in the left primary visual cortex (V1) of two monkeys (Monkeys 3 and 4), trained to perform a fixation task, while visual gratings that varied in size, orientation, contrast or spatial frequency were presented on a screen (see Table 1 and Methods for details). This hybrid grid consisted of 9 (3 × 3) commercially available ECoG electrodes (Ad-Tech Medical Instrument) that are widely used in clinical applications and 81 (9 × 9) microelectrodes (Blackrock Microsystems), both attached to the same connector and referenced to same wire. The microelectrode array was placed between four ECoG electrodes in V1 (see Fig. 1 of ref. ^37^. Receptive field (RF) centers of microelectrode array and ECoG arrays are shown later; for raw traces of the signals and more detailed description of the RFs, see ref. ^37^). For the variable stimulus size condition (Figs. 1–4), data from two additional monkeys (Monkeys 1 and 2) was used, for which microelectrode and ECoG recordings were conducted separately (non-simultaneous recordings, see Table 1 and Methods for details). All spectral analyses were performed using the multi-taper method^38,39^.

**Table 1:** Experimental, stimulus and analysis parameters used in the study.

	Monkey Number	Recording Measure	N	Spatial Frequency (cpd)	Contrast (%)	Radius (degrees)	Orientation (degrees)	AnalysisBaseline Period (ms)	Analysis Stimulus Period (ms)
**Size Study**									
Non-Simultaneous LFP-ECoG Recordings	M1	Spike LFP	15 27	4	Full (~100)	0.3-0.72-1.14-1.56-1.98-2.4	0-30-60-90- 120-150	−200–0	200–400
		ECoG	2	4	Full (~100)	0.3-0.72-1.14-1.56-1.98-2.4	0-30-60-90- 120-150	−200–0	200–400
	M2	Spike LFP	107 71	4	Full (~100)	0.3-0.72-1.14-1.56-1.98-2.4	0-30-60-90- 120-150	−200–0	200–400
		ECoG	1	4	Full (~100)	0.3-0.72-1.14-1.56-1.98	0-30-60-90- 120-150	−200–0	200–400
Simultaneous LFP-ECoG Recordings	M3	Spike LFP	24 77	4	Full (~100)	0.3-0.6-1.2-2.4-4.8-9.6	0-22.5-45-67.5-90- 112.5-135-157.5	−500–0	250–750
		ECoG	5	4	Full (~100)	0.3-0.6-1.2-2.4-4.8-9.6	0-22.5-45-67.5-90- 112.5-135-157.5	−500–0	250–750
	M4	Spike LFP	22 18	4	Full (~100)	0.3-0.6-1.2-2.4-4.8-9.6	0-22.5-45-67.5-90- 112.5-135-157.5	−500–0	250–750
		ECoG	4	4	Full (~100)	0.3-0.6-1.2-2.4-4.8-9.6	0-22.5-45-67.5-90- 112.5-135-157.5	−500–0	250–750
**Orientation Study**									
Simultaneous LFP-ECoG Recordings	M3	LFP	77	4	Full (~100)	Full screen	0-22.5-45-67.5-90- 112.5-135-157.5	−500–0	250–750
		ECoG	5	4	Full (~100)	Full screen	0-22.5-45-67.5-90- 112.5-135-157.5	−500–0	250–750
	M4	LFP	18	2	Full (~100)	Full screen	0-22.5-45-67.5-90- 112.5-135-157.5	−500–0	250–750
		ECoG	4	2	Full (~100)	Full screen	0-22.5-45-67.5-90- 112.5-135-157.5	−500–0	250–750
**Spatial Frequency Study**									
Simultaneous LFP-ECoG Recordings	M3	LFP	77	0.5-1-2-4-8	Full (~100)	Full screen	90	−500–0	250–750
		ECoG	5	0.5-1-2-4-8	Full (~100)	Full screen	90	−500–0	250–750
	M4	LFP	18	0.5-1-2-4-8	Full (~100)	Full screen	90	−500–0	250–750
		ECoG	4	0.5-1-2-4-8	Full (~100)	Full screen	90	−500–0	250–750
**Contrast Study**									
Simultaneous LFP-ECoG Recordings	M3	LFP	77	4	100-50-25-12.5- 6.25-3.125-0	Full screen	90	−500–0	250–750
		ECoG	5	4	100-50-25-12.5- 6.25-3.125-0	Full screen	90	−500–0	250–750
	M4	LFP	18	2	100-50-25-12.5- 6.25-0	Full screen	90	−500–0	250–750
		ECoG	4	2	100-50-25-12.5- 6.25-0	Full screen	90	−500–0	250–750

### High-gamma activity in ECoG is maximum for a small stimulus size (radius of 0.3°)

We first studied the effect of stimulus size. Figure 1A shows the raster plot and multiunit firing rate of an example recording site from Monkey 3 when gratings of six different radii (0.3°, 0.6°, 1.2°, 2.4°, 4.8° and 9.6°) were presented between 0 and 800 ms. The peristimulus histogram averaged across trials is overlaid on each of the raster plots. Consistent with our previous results^17^, increasing the stimulus size decreased the firing rate. Similar trends were observed for the population dataset of 15, 107, 24 and 22 recordings sites from the four monkeys (Fig. 1B; see Table 1 for details).

Next, we studied the LFP and ECoG signals for varying stimulus sizes. Figure 2A shows the mean change in LFP power as a function of time and frequency relative to the baseline period, obtained by subtracting log of baseline power (-500 to 0 ms where 0 is the stimulus onset; see Eq. 1 and Methods for details) for the same example site as Fig. 1A from Monkey 3 for six different sizes. These time-frequency energy difference spectra showed a prominent gamma rhythm (red horizontal band) at ~50 Hz for stimulus size of 0.6° and above, which appeared after the initial transient and remained present throughout the stimulus duration (up to 0.8 s). Consistent with previous studies^16,17,26,29^, strength of LFP gamma rhythm increased with an increase in stimulus size (these effects are quantified in Fig. 3 below) while the gamma peak frequency decreased. Further, the smallest stimulus (radius 0.3°) showed a prominent increase in power over a broad frequency range above the gamma band. The power in this broadband showed the opposite trend and decreased with an increase in stimulus size. Figure 2B shows the time-frequency difference spectra for an example ECoG electrode from the same monkey. Similar to LFP, the power of ECoG gamma increased with increasing stimulus size. Surprisingly, even though the ECoG electrode was much larger than LFP, the smallest stimulus produced the largest high-gamma power even in ECoG. The increase in ECoG high-gamma power was more prominent up to ~250 Hz, unlike LFP high-gamma that remained prominent up to 400 Hz and beyond. Similar results were obtained from the population average of 24 LFP sites and 5 ECoG sites (Fig. 2C,D).

Figure 3A,C,E,G show mean change in power from the baseline (see Table 1 and Methods for baseline ranges), across recording sites, as a function of frequency for Monkeys 1, 2, 3 and 4. In all monkeys, the largest stimulus produced the strongest but slowest gamma, visible as a prominent peak at ~45–60 Hz (orange traces). In all monkeys except Monkey 4, a prominent harmonic of gamma was also visible between 80–120 Hz. However, there were differences between Monkeys 1, 2 and Monkeys 3, 4, potentially because of differences in recording setup and also because much larger stimulus sizes were used for the latter two monkeys. For example, in Monkey 4, a second gamma peak was clearly visible at ~30 Hz for the largest stimulus size, which is the ‘slow’ gamma as described in our previous study that is prominent only for large visual stimuli^29^. Also, the LFP gamma in Monkey 4 was weaker than Monkey 3 (this was also observed in our previous study^29^, in which recordings were done from a different hemisphere using a different array); we discuss this in more detail in the Discussion. Importantly, in spite of the differences in the strength of gamma and high-gamma band across monkeys, the overall trends remained similar: the strength of gamma rhythm increased with an increase in stimulus size whereas high-gamma power decreased. Importantly, similar trends were also observed in the ECoG signals. To compare the changes in power with stimulus size for LFP and ECoG, we computed the power in two frequency bands: 30–65 Hz for gamma and 150–250 Hz for high gamma, as shown in Fig. 3B,D,F,H (raw power was first summed over a frequency band to obtain the total power; the change in this power during stimulus period from baseline was then computed; see Eq. 2 in the Methods section). The gamma range was chosen to avoid the ‘slow’ gamma, while the high-gamma range was chosen to avoid the harmonic of gamma between 80–120 Hz. However, results were similar if a wider gamma range (20–75 Hz) was used (data not shown).

As observed in the change in PSD plots, the power in gamma band increased with size for both LFP and ECoG (the only exception was the ECoG of Monkey 2 for which only a single electrode was available), whereas high-gamma power showed opposite trends. Interestingly, high-gamma power was maximum for the smallest stimulus (radius of 0.3°) for both LFP and ECoG for all the four monkeys. This suggests local origins of ECoG in primary visual cortex, similar to our previous study^37^, since high-gamma would have been expected to be higher for a larger stimulus if spatial summation occurred over a large cortical area for ECoG. However, unlike our previous approaches^37^, this approach did not provide a quantitative estimate of the spatial spread. We discuss this in more detail in the Discussion.

To quantify the similarity in LFP versus ECoG stimulus tuning, we computed the Pearson’s correlation coefficient between the LFP and ECoG change in power values (blue and magenta traces in Fig. 3B,D,F,H; correlation was computed over the six data points for each signal type). The correlations were highly significant in the high-gamma range for all monkeys (Monkey 1: r = 0.85, p = 0.03; Monkey 2: r = 0.99 p = 2.76 × 10^−4^; Monkey 3 r = 0.94, p = 0.005; Monkey 4 r = 0.97, p = 0.001). In the gamma range, the correlations were significant for Monkeys 3 and 4 (Monkey 3: r = 0.99, p = 2.6 × 10^−4^; Monkey 4: r = 0.94, p = 0.01). For the first two monkeys, the correlations were positive but failed to reach significance (Monkey 1: r = 0.74 p = 0.09, Monkey 2: r = 0.61 p = 0.28). This could be because the stimulus size varied over a much smaller range and fewer ECoG electrodes were available for these two monkeys. While these also apply to high-gamma responses (which were significantly correlated in both monkeys), gamma could be more sensitive to the differences in eccentricities^40^ between LFP and ECoG RF centers as compared to high-gamma.

A comparison of the shape of the change in power spectra for LFP (Fig. 3A,C,E,G, top row) versus ECoG (bottom row) revealed an interesting difference. Beyond ~100 Hz, the traces were almost parallel to the x-axis in the case of LFP (in all except Monkey 4) but showed a negative slope for ECoG in all monkeys. This suggested that the slope of the PSD in the high-gamma range during stimulus and baseline periods were comparable in case of LFP (such that the difference produced a zero-slope line), but stimulus PSD had a steeper slope than baseline in case of ECoG. Indeed, we have previously observed that while increase in high-gamma power could be observed up to at least ~400 Hz in LFP^41^, it was prominent only up to ~150 Hz in human ECoG^13^. We quantified this by plotting the slopes of the PSD in the high-gamma range during stimulus period versus baseline. Figure 4A shows the mean PSD and mean slope across electrodes during baseline (black trace, −200 to 0 ms for Monkeys 1 and 2; −500 to 0 ms for Monkeys 3 and 4, where 0 ms is the stimulus onset) and stimulus period (green traces, 200 to 400 ms for Monkeys 1 and 2; 250 to 750 ms for Monkeys 3 and 4) for all four monkeys. As shown in Fig. 4B, the LFP slopes for stimulus and baseline period were comparable (mean slope during stimulus: 1.31, baseline: 1.22, p = 0.15, paired t-test (two sample t-test)), whereas the ECoG slopes for stimulus period were greater than baseline period (mean slope during stimulus: 2.92, baseline: 1.87, p = 0.00035).

### Stimulus tuning of gamma oscillations

Next, we compared the orientation tuning (both preferred angle and selectivity; Eqs. 3 and 4) between LFP and ECoG, for two reasons. First, while it is well established that different neurons prefer different orientations in V1 such that the distribution of orientation preferences of MUA is more or less uniform^42–44^, several studies have shown that the stimulus orientation that generates the strongest gamma in microelectrode recordings is remarkably similar across all the recording sites^16,29,45^. However, since these microelectrode arrays span only ~4 × 4 mm^2^ patch of cortex, it is possible that different patches of cortex prefer different orientations (the preferred orientation for gamma is location specific, but not monkey specific). Because ECoGs record from brain areas separated by 10 mm or more, comparison of orientation preferences across ECoG sites could provide clues about the specificity of orientation tuning in the gamma band. Second, we have recently shown that the orientation selectivity (measure of the strength of orientation tuning) for gamma was much weaker in EEG compared to LFP^29^. This could be because EEG records activity from a much larger part of the brain than LFP, and these parts may not be as well tuned for a particular orientation. A comparison of the orientation selectivity of ECoG and LFP could therefore provide clues about their similarity.

**Figure 5:**
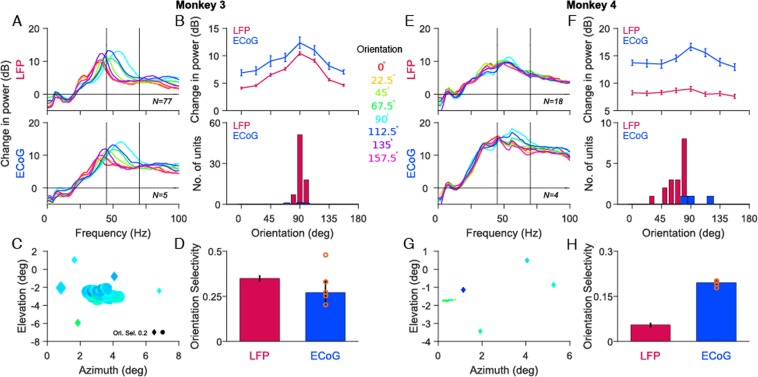
Orientation tuning of gamma oscillations in LFP and ECoG. (**A**) Average relative change in power spectra between 250 and 750 ms from baseline energy (−500 to 0 ms) for 77 LFP (top panel) and 5 ECoG recording sites (bottom panel) for Monkey 3. Eight colored traces are for eight different orientation values (labelled at the centre of Figure). **(B)** Average change in gamma power as a function of orientation (top panel) and the histogram of orientation preference (bottom panel) across recording sites for LFP (magenta) and ECoG (blue). Error bar indicates SEs of the mean. **(C)** Orientation preference of gamma rhythm across LFP (circle) and ECoG (diamond) recording sites plotted at the respective RF centers. The color represents the preferred orientation while the size of the marker represents the strength of tuning. **(D)** Median orientation selectivity of LFP and ECoG across recording sites. Error bar indicates SEs of the median, computed using bootstrapping. The orange circles are the five ECoG electrodes. **(E–H)** same as (**A–D**) but for 18 LFP and 4 ECoG recording sites in Monkey 4.

Figure 5A shows the population average of the change in LFP and ECoG power from baseline as a function of frequency, across 77 LFP (top) and 5 ECoG (bottom) recording sites for Monkey 3. As before, the change in power was computed between 250 ms to 750 ms relative to baseline period (0 ms to 500 ms before stimulus onset) and then averaged across sites on a log scale. The eight colored traces represent the change in power spectrum for eight stimulus orientations. We observed that the mean LFP gamma between 45 to 70 Hz was strongest and fastest at a stimulus orientation of 90°. Surprisingly, mean ECoG gamma showed similar trends as LFP gamma with the strongest and fastest gamma for 90° orientation (Fig. 5B, top panel; r = 0.99, p = 2.84 × 10^−8^).

To examine the preferred orientation of gamma at different cortical locations we computed the preferred orientation of gamma in 45 to 70 Hz frequency range for each of the recording sites. Figure 5C shows ECoG (diamonds) and LFP (circles) electrodes, plotted at their receptive field centers and color-coded based on preferred orientation for Monkey 3. Consistent to previous studies^16,29,45^, we observed that preferred orientation of LFP gamma was similar across sites (Fig. 5B, bottom panel, magenta bars). Interestingly, all the five ECoG electrodes which covered ~20 × 20 mm in the cortex, showed a remarkably similar preference for stimulus orientation. Although we observed small variations in preferred orientation from the electrode to electrode, the distribution of ECoG (ranging from 70° to 100°) was similar to the LFP (ranging from 80° to 100°; Fig. 5B, bottom panel). Further, the strength of orientation tuning measured by orientation selectivity was on average comparable for ECoG and LFP (Fig. 5D). The ECoG electrodes which showed a deviation from 90° had low orientation selectivity values, represented by the smaller marker size in Fig. 5C. Similar results were observed for Monkey 4 across 18 LFP and 4 ECoG recording sites, albeit the correlation was weaker because gamma was less salient in this monkey (Fig. 5E–H; Pearson correlation coefficient of 0.68, p = 0.06). Thus, the orientation preference of gamma is monkey specific but not location specific.

The orientation preference and selectivity depended on the choice of the frequency band. In particular, for Monkey 3, gamma peak frequency was below our lower cutoff of 45 Hz for some orientations. We used this gamma range to be in congruence with our previous study^29^, in which we had recorded from the same monkeys but used a microelectrode array implanted in the other hemisphere, and had also collected simultaneous EEG data. Since the orientation preferences for LFPs were similar for the two arrays, having the same frequency range allowed us to better compare the LFP, ECoG and EEG gamma tuning. Further, the low frequency cutoff could not be lowered due to the presence of ‘slow gamma’ (see ref. ^29^), which peaked between 30–35 Hz for the two monkeys. As discussed in more detail later, tuning properties critically depend on the choice of the lower frequency cutoff. Nonetheless, visual inspection of Fig. 5A,E reveals that the gamma peaks were remarkably similar for LFP and ECoG for both monkeys, such that choosing a different frequency range changed the tuning parameters in similar ways. To verify this, we used a broader frequency range of 20–75 Hz to accommodate the gamma peaks across all the orientation values, but instead of taking the average power over this band (which is dominated by lower frequencies within the band), we computed the frequency at which the gamma peak was observed in the baseline corrected PSD and used the value at that frequency (Supplementary Fig. 1). Both the gamma peak frequency (Supplementary Fig. 1,B,D, top panel) and change in power at peak frequency (B and D, bottom panel) changed in similar ways as a function of stimulus orientation for LFP and ECoG (Monkey 3: Gamma peak: r = 0.86, p = 0.01; power: r = 0.89, p = 0.01; Monkey 4: Gamma peak: r = 0.72, p = 0.05; Power: r = 0.76, p = 0.03). As in Fig. 5, tuning was weaker for Monkey 4.

We also repeated the analysis shown in Fig. 5 after taking bipolar referencing (see Methods for details), which reduces any component in the signal that is common to both electrodes (Supplementary Fig. 2). Indeed, slow gamma diminished under this scheme, consistent with the idea that it is a more global signal than fast gamma (see ref. ^29^ for details). Importantly, results remained similar after bipolar referencing (Monkey 3: r = 0.99, p = 1.72 × 10^−6^; Monkey 4: r = 0.76, p = 0.03), suggesting that our results were not affected by the choice of referencing scheme.

**Figure 6:**
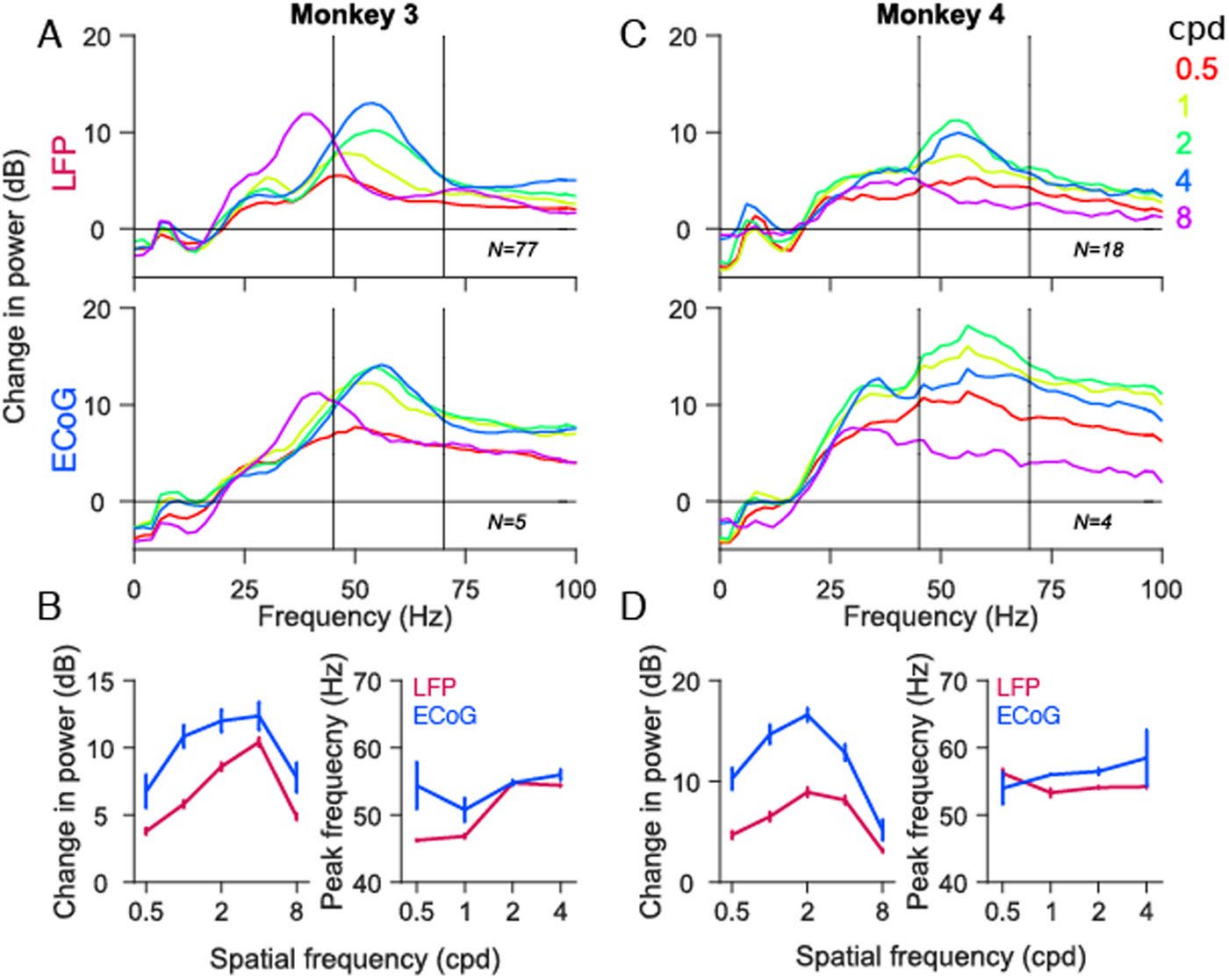
Spatial frequency tuning of gamma oscillations in LFP and ECoG. **(A,C)** Mean change in power spectra across 77 and 18 LFP recording sites (top panel), 5 and 4 ECoG recording sites (bottom panel) for Monkeys 3 and 4 calculated at stimulus orientationss that induce largest power change in gamma (90° for both monkeys). Five colored traces represent five different spatial frequency values. **(B,D)** left panel: Average change in gamma power as a function of spatial frequency for LFP (magenta) and ECoG (blue). right panel: Average gamma peak frequency as a function of spatial frequency. 8 cpd was ignored as the gamma peak was out of the selected frequency range. Error bar indicates SEs of the mean.

Next, we computed the tuning preferences of LFP and ECoG gamma for spatial frequency. Figure 6A,C show the change in power (relative to baseline as before, top panel), averaged across recording sites for Monkey 3 and Monkey 4. Consistent with previous studies^16,29^, power in LFP gamma was higher between 1 and 4 cpd than 0.5 and 8 cpd (in Monkey 3, the 8 cpd stimulus produced a gamma peak in the slow gamma range in both LFP and ECoG; consistent with our previous study in which an array was inserted in the other hemisphere of this monkey^29^). The strength of LFP gamma was maximum for 4 and 2 cpd for Monkeys 3 and 4. Similar trends were observed for ECoG gamma (Fig. 6A,C, bottom panel). As before, we computed the change in gamma power in the 45 to 70 Hz frequency range to avoid the contributions from ‘slow gamma’ band which peaked around ~30 Hz and ~36 Hz in Monkeys 3 and 4 (Eq. 2; Fig. 6B,D). Both LFP and ECoG gamma power showed similar trends (Monkey 3: r = 0.91, p = 0.03; Monkey 4: r = 0.91, p = 0.03). Gamma peak frequency, defined as the frequency within the considered band at which the change in power was maximum, was similar for the different spatial frequency values for both LFP and ECoG, leading to insignificant correlation values between them (Monkey 3: r = 0.68, p = 0.32; Monkey 4: r = −0.63, p = 0.37; 8 cpd was not considered because the center frequency was out of the specified range).

**Figure 7:**
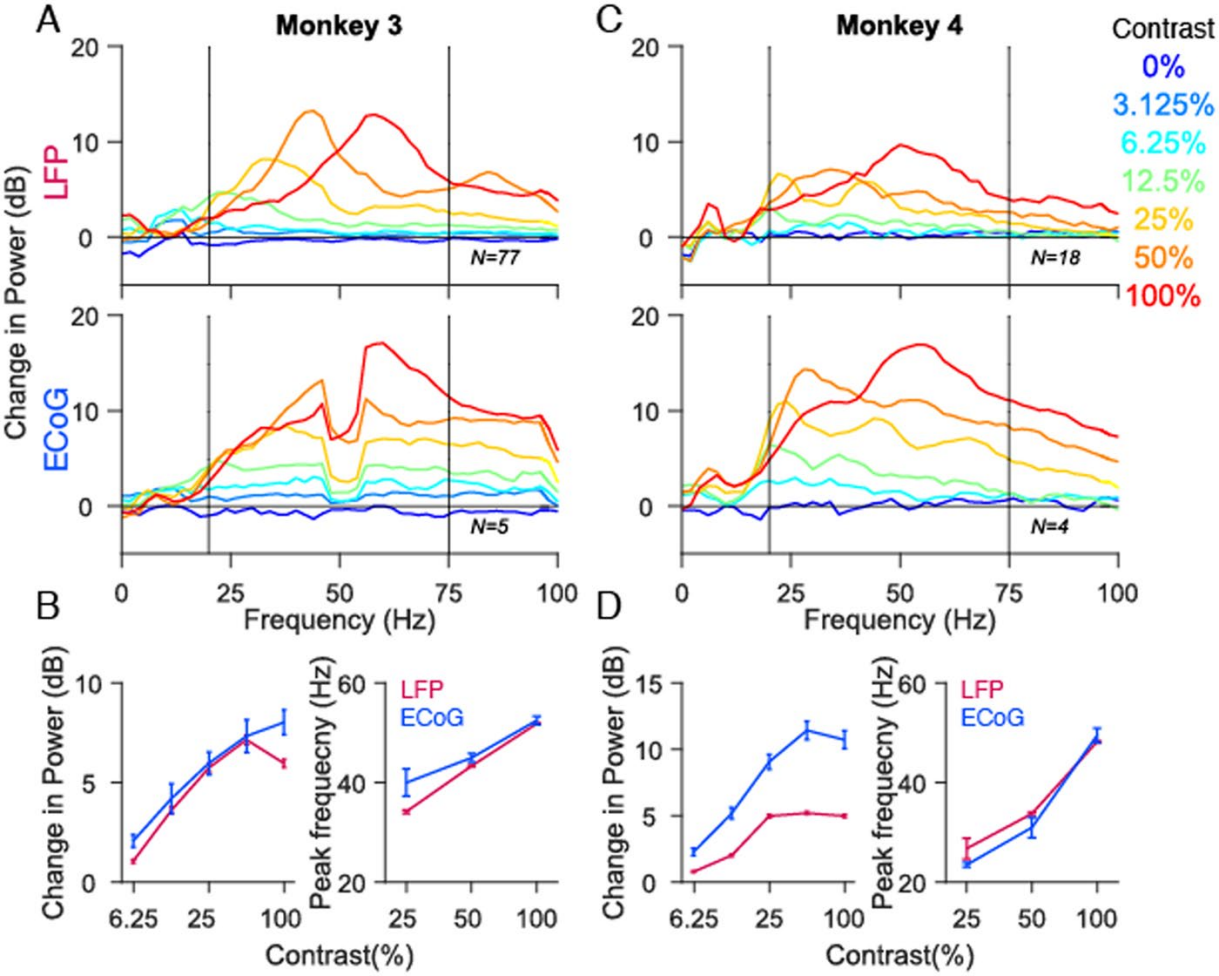
Contrast tuning of gamma oscillations in LFP and ECoG. **(A,C)** Mean change in power spectra across 77 and 18 LFP recording sites (top panel), 5 and 4 ECoG recording sites (bottom panel) for Monkeys 3 and 4 calculated at stimulus orientations and spatial frequencies that induce largest power change in gamma (90° and 4cpd for Monkey 3 and 90° and 2cpd for Monkey 4). Seven colored traces represent seven different contrast values. Note that for Monkey 4 there are only six traces. **(B,D)** left panel: Average change in gamma power as a function of contrast for LFP (magenta) and ECoG (blue). right panel: Average gamma peak frequency as a function of contrast. Error bar indicates SEs of the mean.

Finally, we computed the tuning preference of gamma in LFP and ECoG for stimulus contrast. Consistent with several previous studies^25,27–29,46–49^, gamma power as well as peak frequency increased with stimulus contrast in both LFP and ECoG (Fig. 7; one study (ref. ^25^) had reported an increase in power but no increase in peak frequency with contrast, but that is likely due to the use of small stimuli that produced a weak gamma rhythm). To quantify this, we computed the change in gamma power in 20 to 75 Hz frequency range to accommodate the lower stimulus contrasts for which gamma peaked at lower frequencies. The change in gamma power initially increased with stimulus contrast and plateaued at higher contrasts for both LFP and ECoG (Fig. 7B,D left panel; Monkey 3: r = 0.95, p = 0.01; Monkey 4 r = 0.98, p = 0.003). Similarly, LFP and ECoG gamma peak frequency increased with contrast (Fig. 7B,D right panel; Monkey 3: r = 0.99, p = 0.09; r = 0.99 p = 0.03; only 25%, 50% and 100% are used because the gamma peaks are salient only at these contrasts). It should be noted that ECoG recordings in Monkey 3 showed prominent line noise at 50 Hz and therefore the peak frequency computation might not be accurate for 50% and 100% stimulus contrast values. Nevertheless, an increase in peak frequency with contrast can be observed by visual inspection of the traces. For computing the change in gamma power, we ignored the frequency values at which line noise was observed.

## Discussion

We compared the stimulus tuning properties of gamma/high-gamma in LFP and ECoG by simultaneously recording these signals using a custom-made hybrid grid and found them to be surprisingly similar. The smallest stimulus size tested (radius of 0.3°), which has been earlier shown to produce largest high-gamma power in LFP^25,27–29,45^, produced the largest high-gamma power in ECoG as well. Further, tuning preferences of gamma oscillations for stimulus size, orientation, spatial frequency and contrast were very similar for LFP and ECoG. Overall, these results suggest that ECoG is a local signal as well as an excellent signal to study gamma oscillations.

These results are consistent with our recent study^37^, in which we used a receptive field (RF) mapping approach to show that the spatial spread of ECoG was surprisingly local (SD of ~1.5 mm or 2 SD of ~3 mm), not much larger than the diameter of the ECoG electrode (2.3 mm), and only ~3 times the spread of LFP (2 SD of ~ 1 mm). These results are also consistent with the observation that the RFs of ECoGs recorded in humans are very small^3^, although in that study the RFs (measured in degrees) were not converted to cortical spreads (measured in mm).

Unfortunately, the approach used in the current study did not yield a quantitative estimate of the ECoG spread, for two reasons. First, it is possible that ECoG preferentially samples neurons in the upper layers of the cortex that may prefer smaller stimulus sizes, so it is difficult to deduce spatial spread only from size tuning. Second, the range of stimulus sizes that we used was not wide enough to quantitatively compare the spreads of LFP and ECoG. Use of even smaller stimuli (for example, radius of 0.1°) would have yielded a better estimate of the ‘optimal’ stimulus size for LFP high-gamma power, and comparison of optimal stimulus sizes for LFP and ECoG would have yielded a quantitative estimate of their respective spatial spreads. However, when extremely small stimuli are used, appropriate comparison is possible only in the absence of eye jitters. Given that the monkey had to maintain fixation only within 1° (Monkeys 1 and 2) or 2° (Monkeys 3 and 4) around the fixation spot, it is possible that a very small stimulus would occasionally miss the receptive field completely if the monkey’s gaze was away from the fixation spot, increasing the variability of the estimate of high-gamma power for very small stimuli. The method used in our previous study^37^, which is originally based on the model proposed by Xing and colleagues^50^, partially addressed this concern because the inflation in the estimate of the RF size due to several factors (including eye jitters) is similar for different measures (MUA, LFP and ECoG), and therefore a model that estimates the spatial spreads based on the differences in RF sizes between measures (such as MUA versus LFP and LFP versus ECoG) can cancel out these common terms (see refs. ^37,50^ for details). We had also used another approach that involved the comparison of the PSDs of ECoG and LFP during spontaneous periods to show that the ECoG spread was local. The present approach, obtained by simply comparing the high-gamma power as a function of stimulus size, provides a third, albeit weaker line of evidence that ECoG is a local signal. However, this result is obtained without any model or additional assumptions and is complementary to the previous two approaches that used either very small stimuli to map RFs or compared the PSDs during spontaneous periods. Further, we add to our previous study^37^ by showing that the tuning preferences of LFP and ECoG are very similar across a wide range of stimulus properties (size, orientation, spatial frequency and contrast), which also points to local origins of ECoG.

What are the origins of high-gamma activity in ECoG? High-gamma activity was initially interpreted in the same conceptual framework as gamma oscillations, just operating at a higher frequency^51–53^. More recently, high-gamma in the LFP has been shown to be tightly correlated with the multiunit firing rate^13–17^. ECoG high-gamma power has been proposed to reflect the synchrony in neural population^13^, although direct experimental evidence, to our knowledge, is lacking. In the size study, we observed that upper range of ECoG high-gamma was limited to 200–250 Hz compared to at least 400 Hz in LFP (see Fig. 2B vs 2A for stimulus radius of 0.3°). This was consistent across electrodes (Fig. 2D vs 2C) and monkeys (Fig. 3A,C,E,G; bottom vs top panel), and was further quantified by comparing the slopes in stimulus period with baseline period (Fig. 4). This could be because the PSD of the ECoG was much steeper than LFP at low frequencies (see ref. ^37^), and therefore the overall power of the ECoG at high frequencies was much lower than LFP. Thus, the noise (either in the device or the brain) could have affected the ECoG signal more than LFP at high frequencies. It appears that even the LFP for Monkey 4 was more affected by noise, since the PSD slopes in this monkey were shallower during both baseline and stimulus periods compared to other monkeys (Fig. 4). The differences in PSD slopes for ECoG compared to LFP could be due to its larger size, lower impedance, or position.

Characterization of the PSD slope is important because it may reveal information about the underlying cortical processing and could potentially be used as a marker in understanding cognitive states in human brain disorders^54,55^. Consistent with previous ECoG studies in humans^55,56^,we also observed the ECoG PSD slopes in the range of 1 to 3. More recently, some studies have shown a tilt in the spectrum or a decrease in slope in 10–100 Hz upon activation^56^ and in the high-gamma range (80–150 Hz) with ageing^55^. Consistent with this, visual inspection of the ECoG PSDs in Fig. 4A (bottom row) indeed shows that the stimulus PSD slope becomes shallower than the baseline PSD at low frequencies (<100 Hz; most prominent in Monkeys 3 and 4) before becoming steeper in the high-gamma range as quantified in Fig. 4B. Unfortunately, because we used gratings that induced narrowband gamma oscillations between 20–70 Hz, it is difficult to decouple the effect of gamma rhythm from PSD slope estimation below 100 Hz in our data (Podvalny and colleagues had used a visuomotor task in which faces were shown, which induced a gamma peak around ~80 Hz and above). PSD flattening was not evident in the LFPs (Fig. 4A, top row), consistent with a previous study^41^. The flattening of the PSD observed by Podvalny and colleagues could be related to the cognitive load, since in their study subjects performed a visuo-motor or object recognition task rather than a simple fixation task.

We observed that the tuning preferences of gamma were similar for ECoG and LFP for all the four stimulus manipulations (size, orientation, spatial frequency and contrast), while previously we had observed considerable differences between LFP and EEG tuning^29^. Note that while these recordings were done on the same monkeys, we did not record all three signals simultaneously because of technical difficulties (see Methods). Nonetheless, the weak tuning of EEG gamma was observed in humans also^29^, and is therefore likely to be a general feature of EEG signals. The similarity in tuning profile of LFP and ECoG gamma rhythms for different stimulus manipulations could be because of a coherent network because of the use of full screen gratings at full contrast which are known to produce strong and coherent gamma rhythms^16,17^ over a large brain area. Both the microelectrodes and macroelectrodes captured the activity of this network and therefore showed similar tuning preferences. Interestingly, ECoG electrodes which were on the surface of cortex captured this activity as reliably as microelectrodes which were presumably in layers 2/3 of the cortex. Apart from the stimulus, another factor that could have influenced our results is volume conduction^57,58^. In a previous study^58^, in which we recorded from microelectrodes implanted in Monkeys 1 and 2, we showed that the LFP-LFP phase coherence almost becomes flat for CSD (current source density, a double spatial derivative of potential, obtained by subtracting the potential of an electrode from the potentials of four neighboring electrodes; see Fig. 4A of ref. ^58^). Since, we had only five (Monkey 3) and four (Monkey 4) ECoG electrodes, the CSD analysis could not be performed for ECoG in the current setup. We were, however, able to perform bipolar referencing, which did not alter the tuning preferences in LFP or ECoG (Supplementary Fig. 2).

The simultaneous recordings in Monkeys 3 and 4 used a common reference wire for microelectrode and macroelectrode (ECoG) recordings and therefore it can be argued that the similar tuning preferences of LFP and ECoG are because of a shared reference. However, this is unlikely since the RFs (estimated by presenting a small stimulus at various positions in the visual field) were well separated for micro and macro electrodes (see Fig. 3 of ref. ^37^). If the recordings were driven by a common shared reference the RFs would have been similar and overlapping, which was not the case. Similarly, in the size study, small stimuli that covered the RFs of either the micro or the macro electrodes did not produce any response in the other signal. Further, LFP and ECoG tuning profiles were similar to Fig. 5 after bipolar referencing, suggesting that the similarity in gamma tuning preference was not because of a common reference.

As described earlier in Results section, the tuning parameters depended critically on the low frequency limit of the gamma band. This is because the actual power (not change in power which is displayed in the figures) falls off rapidly with frequency and displays a prominent “1/f” structure. The total power in a band is therefore dominated by the lower frequencies that have larger absolute power. For example, in the orientation tuning experiment, gamma peak was strongest for the stimulus orientation of 90° but also the fastest (peak around ~55 Hz) for Monkey 3 (Fig. 5A). Orientation of 0° produced a smaller bump, but since it was around 40 Hz, the power between 35–40 Hz was more for 0° stimulus than 90°. However, if we had chosen the gamma band between 35–70 Hz, the preferred orientation would have shifted towards 0° just because the absolute power between 35–40 Hz far exceeds the power between 50–60 Hz. This issue can be partially addressed by using the normalized instead of absolute power while computing the power in a band, or by using the power at which gamma peak is observed (as shown in Supplementary Fig. 1). However, in general, it is difficult to compare gamma power across stimulus conditions when the peak frequency itself shifts with stimulus. In our case, the choice of frequency band is of less relevance because the actual power spectra for LFP and ECoG were remarkably similar for every stimulus condition: if the gamma peak did not fall in a specified range for LFP, it invariably fell outside the range for ECoG as well. Therefore, our main result that LFP and ECoG gamma tuning is remarkably similar holds irrespective of the choice of the frequency band.

Although the overall trends were similar for Monkeys 3 and 4, the strength of tuning was different. This is not surprising, since in our previous recordings as well we have observed considerable variability across animals. In particular, we have recorded from the other hemisphere of Monkeys 3 and 4 using different arrays^29^ and found that the selectivity is weaker for Monkey 4 versus 3 even in that dataset (see Figs. 1 and 2 of ref. ^29^), ruling out the possibility that poor selectivity in Monkey 4 was due to poor data quality. Importantly, the orientation selectivity for both monkeys across the two implants remained similar, again suggesting that orientation selectivity in monkey specific, not location specific. In general, while there is variability in gamma responses across monkeys, these responses remain highly conserved across sessions within a monkey, even when separated by several months (data not shown). We have also recently recorded grating-induced gamma from human subjects using EEG and observed large variability in both slow and fast gamma power in healthy population, even in age and gender matched data^59^. This variability across subjects does not affect any of the results shown here because we compared the tuning preferences of gamma in two signals (LFP and ECoG) from the same individual.

To conclude, our findings highlight the presence of gamma oscillations in ECoG which shows similar tuning preference to gamma oscillations observed in LFP recordings, even though the size of the ECoG electrode is several hundred times larger than the microelectrode. Further, comparing the high-gamma activity between ECoG and LFP we showed that ECoG has local origins in V1. Together, our results validate the use of ECoG in brain-machine interface applications and basic science research.

## Methods

### Animal preparation and recording

All animal experiments and protocols performed in this study are in strict accordance with the relevant guidelines and regulations approved by the Institutional Animal Care and Use Committee of Harvard Medical School (for Monkeys 1, 2) and Institutional Animal Ethics Committee (IAEC) of the Indian Institute of Science and the Committee for the Purpose of Control and Supervision of Experiments on Animals (CPCSEA) (for Monkeys 3 and 4). The details of our experiment design and data collection have been described in detail in our previous study^37^; here we explain them briefly. The microelectrode and ECoG data used in this study were collected in two separate set of experiments. The first set was conducted on two male monkeys (*Macaca mulatta*; 11 and 14 Kg); animal protocols approved by the Institutional Animal Care and Use Committee of Harvard Medical School. For this set of experiments, microelectrode and ECoG recordings were performed separately and are described in detail elsewhere^17,27,60^. Briefly, after monkeys learned the behavioral task, a 10 × 10 microelectrode grid (96 active channels, Blackrock Microsystems) was implanted in the right primary visual cortex (~15 mm anterior to the occipital ridge and ~15 mm lateral to the midline). The microelectrodes were 1 mm long separated by 400 μm. After microelectrode recordings, a second surgery was performed to implant the custom-made array having 2 ECoG contacts (2.3 mm in diameter and 10 mm apart, Ad-Tech Medical Instrument, the standard size used in clinical applications) on the left primary visual cortex of the same monkeys (see Materials and Methods of ref. ^37^, for details). One ECoG electrode in Monkey 2 did not show any stimulus evoked response and thus was excluded, yielding 3 ECoG electrodes from these two monkeys. Note that ECoG and microelectrode recordings were non-simultaneous for these two monkeys. However, we have observed in chronic recordings that are active for long periods of time (several months) that tuning preferences do not change over time. Therefore, we do not expect the non-simultaneity of LFP and ECoG recordings in these two monkeys to influence any of the results.

The second set of experiments involved simultaneous recordings of spikes, LFP and ECoG signals from two female adult monkeys (*Macaca radiata*; 3.3 and 4 Kg); animal protocols approved by the Institutional Animal Ethics Committee (IAEC) of the Indian Institute of Science and the Committee for the Purpose of Control and Supervision of Experiments on Animals (CPCSEA). Once the monkey had learned the fixation task, a custom-made hybrid array (see Fig. 1 of ref. ^37^) was implanted in the left cerebral hemispheres. This hybrid array had 3 × 3 ECoG electrodes (Ad-tech Medical Instrument; same size as used in Monkeys 1 and 2 and used worldwide in clinical applications) and 9 × 9 microelectrodes, both attached to the same connector made by Blackrock Microsystems. The ECoG electrodes were platinum discs of exposed diameter of 2.3 mm and inter-electrode center- to-center distance of 10 mm. The microelectrodes were 1 mm long, 400 μm apart. The electrode array was implanted under general anesthesia; first a large craniotomy and a smaller durotomy were performed, subsequent to which the ECoG sheet was inserted subdurally such that the previously made silastic gap between four ECoG electrodes was in alignment with the durotomy (see ref. ^37^ for details). The microelectrode array was finally inserted into the gap, ~10–15 mm from the occipital ridge and ~10–15 mm from the midline. In Monkey 3, out of six ECoG electrodes which were posterior to lunate sulcus, one had noisy receptive field estimate, yielding 5 ECoG electrodes for further analysis. For Monkey 4, the ECoG grid did not slide smoothly on the cortex and one column (electrodes 1–3) had to be removed, yielding 4 ECoG electrodes in V1. Two reference wires, common for both microelectrode and ECoG grid were either inserted near the edge of the craniotomy or wounded over the titanium screws on the metal strap which was used to secure the bone on the craniotomy. Other findings based on data recorded from Monkeys 3 and 4 but from a different microelectrode array (implanted in the right hemisphere) have been reported elsewhere.

In case of Monkey 4, we used a hybrid array that had been implanted on a different monkey, but it had to be explanted after 2 days due to complications related to the surgery. One reference wire was lost during the process, and the insulation was removed from the other one (in Monkey 3, insulation from only the tip of the reference wires were removed). This could have led to higher noise in the LFP data collected from Monkey 4 at frequencies above 250 Hz, because the power spectral density appeared to be shallow than other monkeys. It is unlikely that this affected any of the results, since clear gamma rhythm and high-gamma activity were observed in the LFP, which were generally similar to the recordings done earlier using a fresh array implanted in the other hemisphere^29^. Further, ECoG electrodes that were simply placed on the cortex were unaffected by the explantation and showed strong gamma peaks.

All signals were recorded using Blackrock Microsystems data acquisition system (Cerebus Neural Signal Processor). Local field potential (LFP) and multi-unit activity (MUA) were recorded from microelectrode array. LFP and ECoG were obtained by band-pass filtering the raw data between 0.3 Hz (Butterworth filter, first order, analog) and 500 Hz (Butterworth filter, fourth order, digital), sampled at 2 kHz and digitized at 16-bit resolution. MUA was derived by filtering the raw signal between 250 Hz (Butterworth filter, fourth order, digital) and 7,500 Hz (Butterworth filter, third order, analog), followed by an amplitude threshold (set at ~6.25 (Monkey 1), ~4.25 (Monkey 2) and ~5 (Monkeys 3 and 4) of the SDs of the signal).

The data acquisition system has provisions to measure both the impedance of the electrodes as well as potential cross-talk across pairs of electrodes. The similarity in the gamma oscillations recorded in LFP and ECoG signals was not due to potential crosstalk between LFP and ECoG electrodes, which we could measure explicitly. Further, RF centers for LFP and ECoG electrodes were far apart (Fig. 5C,G), and small stimuli that covered the RF of only one signal produced salient gamma oscillations in that signal but virtually no response in the other, ruling out potential cross-talk influencing our results.

Previously we had also recorded EEG data from Monkeys 3 and 4 simultaneously with the LFP^29^. In this study, EEG signals were found to be extremely noisy. This was because a much larger craniotomy was needed to insert the ECoG array, and consequently a larger titanium mesh, longer plates and more screws were required to secure the bone flap. Further, as this was the second surgery on these monkeys, there was considerable hardware present on the other hemisphere from the first surgery as well. Consequently, there was hardly enough space to put EEG electrodes on the occipital areas, and those signals were noisy.

### Behavioral task

Three separate datasets were used in this study. The first set was used to study the effect of size (‘size study’, Figs. 1–4) on LFP and ECoG power and were collected from all four monkeys. The second and third data sets were collected from Monkeys 3 and 4 to study the effect of orientation and spatial frequency (‘orientation and spatial frequency study’, Figs. 5 and 6) and the effect of contrast (‘contrast study’, Fig. 7) on LFP and ECoG power. The behavioral task and stimuli used in these studies are described below in detail.

#### Size study

The data set and results from microelectrode recordings from the first two monkeys have been reported previously^17^. The experimental design and behavioral task for ECoG recordings were similar. Monkeys 1 and 2 performed an orientation change task, while two achromatic odd-symmetric stimuli were presented synchronously for 400 ms with an inter-stimulus period of 600 ms. A Grating stimulus of variable size centered on the receptive field of one of the recording electrodes (new location for each session) was presented in the left hemifield for microelectrode recordings and right hemifield for ECoG recordings. The monkeys maintained fixation within 1° of a small fixation dot (0.05 to 0.10° diameter) and were cued to attend to a low-contrast Gabor stimulus outside of the receptive field (RF) and respond to a change in the orientation of the Gabor stimulus by 90° in one of the presentations. Monkeys responded by making a saccade within 500 ms of the orientation change. The Gratings were a static stimulus with a spatial frequency of 4 cycles/degree (cpd), full contrast, located at the center of the RF of one of the sites (different recording site each session), one of six different orientations (0°, 30°, 60°, 90°, 120° and 150°) and six different radii (0.3°, 0.72°, 1.14°, 1.56°, 1.98° and 2.4°), chosen pseudo-randomly. For ECoG recordings in Monkey 2, only five radii were presented (up to 1.98°), since the RF center of the ECoG electrode was very fovial (azimuth: 1.16, elevation: 1.83) and the largest stimulus (2.4°) covered the fixation spot. The Gabor stimulus presented outside the RF was also static with an SD of 0.5°, spatial frequency 4 cpd and an average contrast of ~6% and ~4.3% for Monkeys 1 and 2. Monkeys 1 and 2 performed the task in 10 and 24 recording sessions for microelectrode recordings (results presented in ref. ^17^; and 2 and 1 recordings sessions for ECoG recordings (one session for each ECoG electrode).

Monkeys 3 and 4 performed the fixation task while they were in a monkey chair, with their head fixed by the headpost. The monkeys were required to hold their gaze within 2° of a small central dot (0.10° diameter) located at the center of a monitor (BenQ XL2411, LCD, 1280 × 720 pixels, dimensions of 53.13 × 29.88 cm, 100 Hz refresh rate, gamma corrected) and were rewarded with a juice pulse at the end of the trial upon successful fixation. The stimulus was a Grating with a spatial frequency of 4 cpd, full contrast, one of eight different orientations (0°, 22.5°, 45°, 67.5°, 90°, 112.5°, 135° and 157.5°) and six different radii (0.3°, 0.6°, 1.2°, 2.4°, 4.8° and 9.6°), chosen pseudo-randomly, presented for 800 ms with an inter-stimulus period of 700 ms at the RF of one of the recording sites (different recording site each session). The data were collected in 15 (Monkey 3) and 6 (Monkey 4) recording sessions for microelectrode recordings and 5 (Monkey 3) and 4 (Monkey 4) recording sessions for ECoG electrodes.

Only correct trials were used for analysis. For each stimulus size condition, the trials were pooled across orientations to increase the statistical power. The average number of repetitions for each size condition for LFP and ECoG were 182 (range 133 to 288) and 141 (range 129 153) for Monkey 1, 145 (range 106 to 196) and 176 (range 173 to 179) for Monkey 2, 79 (range 37 to 205) and 150 (range 92 to 189) for Monkey 3, and 91 (range 30 to 127) and 115 (range 87 to 153) for Monkey 4.

#### Orientation and spatial frequency tuning study

A full-screen static Grating (which subtended a visual angle of ~56° and ~33° in horizontal and vertical directions) stimulus was presented for 800 ms with an inter-stimulus period of 700 ms while Monkeys 3 and 4 performed a fixation task. The Gratings were presented at full contrast at one of five spatial frequencies (0.5, 1, 2, 4 and 8 cpd) and one of the eight orientations (0°, 22.5°, 45°, 67.5°, 90°, 112.5°, 135° and 157.5°) chosen pseudo-randomly. The effect of orientation was studied (Fig. 5) at spatial frequency which produced highest power in gamma range (4 and 2 cpd for Monkeys 3 and 4). The average number of repetitions for each orientation condition and preferred spatial frequency were 33 (range 28 to 36) for Monkey 3 and 42 (range 37 to 45) for Monkey 4. Similarly, the effect of spatial frequency was studied (Fig. 6) at preferred orientation (~90°) which produced highest gamma power. The average number of repetitions were 33 (range 32 to 36) and 34 (range 15 to 45).

#### Contrast study

The stimulus for Monkey 3 was a full-screen Grating at preferred spatial frequency (4 cpd), preferred orientation (90°), one of seven contrasts (100, 50, 25, 12.5, 6.25, 3.125 and 0%) and one of eight different temporal frequencies (tf = 50, 32, 16, 8, 4, 2, 1and 0 cycle per second; counterphase). We studied (Fig. 7) the effect of contrast for the static grating (tf = 0 cps); average number of repetitions was 17 (range 16 to 18). For Monkey 4, stimulus was a static full screen Grating at preferred spatial frequency (2 cpd), one of the six contrasts (100, 50, 25, 12.5, 6.25 and 0%) and one of the eight orientations (0°, 22.5°, 45°, 67.5°, 90°, 112.5°, 135° and 157.5°). Contrast tuning was studied at preferred orientation (90°); average number of repetitions was 27 (range 26 to 29). Both monkeys performed a fixation task and stimulus was presented for 800 ms with an inter-stimulus period of 700 ms.

### Electrode selection

Receptive fields were mapped by flashing small Gabor stimuli at various positions on the screen, as described in detail in our previous studies^37,60^. As in our previous studies, only electrodes for which the RF estimates were stable across days (SD less than 0.1°) were used for further analysis, yielding 27, 71, 77 and 18 microelectrodes and 2, 1, 5 and 4 ECoG electrodes from Monkeys 1, 2, 3 and 4.

For the size study, the smallest stimulus was of radius 0.3°, covering only a few microelectrodes in the visual field. Therefore, for each recording session, we selected electrodes whose RF centers were within 0.2° of the stimulus center. Since we recorded multiple sessions, the same electrode was counted more than once, yielding 56 (24 unique), 141 (66 unique), 62 (40 unique) and 70 (18 unique) electrodes for Monkeys 1–4. Out of this set, we selected electrodes for which the average firing rate was at least 1 spike/s (for an analysis period of 200 to 400 ms for Monkeys 1 and 2 and 250 to 750 ms for Monkeys 3 and 4) for all the stimulus sizes, and a signal-to-noise ratio^61^ greater than 1.5. This yielded 15 (11 unique), 107 (58 unique), 24 (20 unique) and 22 (13 unique) electrodes for further analysis for the four monkeys.

For the orientation, spatial and contrast studies, full screen stimuli were used because that condition produced the strongest gamma. Consequently, firing rates were weak for most sites^29^. Since our primary interest was to compare gamma power, we used the full set of 77 (Monkey 3) and 18 (Monkey 4) microelectrodes and compared the power with 5 (Monkey 3) and 4 (Monkey 4) ECoG electrodes.

### Data analysis

All the data were analyzed using custom codes written in MATLAB (The MathWorks, RRID:SCR_001622). Power spectral density (PSD) and the time-frequency spectra were computed using the multi-taper method with three tapers, implemented in Chronux 2.0 (Bokil *et al*., 2010, RRID:SCR_005547), an open-source, data analysis toolbox available at http://chronux.org. The baseline period was chosen between −200 to 0 ms for Monkeys 1 and 2 and −500 to 0 ms for Monkeys 3 and 4, where 0 indicates stimulus onset. Stimulus period was chosen between 200 to 400 ms for Monkeys 1 and 2 and 250 to 750 ms for Monkeys 3 and 4 to avoid the stimulus-onset related transients.

Time-frequency difference spectra shown in Fig. 2 were obtained by first computing the time-frequency power spectra using a moving window of size 250 ms and a step size of 25 ms and then subtracting the baseline power:

1 $$D(t,w)=10\times (lo{g}_{10}E(t,w)-lo{g}_{10}B(w))$$

where $E(t,w)$ is the mean energy averaged over trials at time t and frequency w, and $B(w)$ is the baseline energy computed for 500 ms (−500 to 0 ms before stimulus onset). Since subtraction is done on a log scale, this is essentially the log of the ratio of power at any time and the baseline power and has units of decibel (dB). For population data (Fig. 2C,D), the $D(t,w)$ values over recording sites were averaged. Note that the baseline energy was calculated across all the stimulus conditions for each recording site.

For the size study, gamma range was chosen between 30–65 Hz for all the four monkeys (Fig. 3). This was done to accommodate the peak frequency for all stimulus sizes, as gamma peak frequency decreases with an increase in stimulus size^17,28,29^. The high-gamma range (150–250 Hz) was chosen higher than usual (>80 Hz) to avoid the harmonic of gamma rhythm (~100 Hz, see Fig. 3). The gamma frequency range for orientation and spatial frequency studies, in which a full-screen Grating was presented, was chosen to be 45–70 Hz for Monkeys 3 and 4. This was done in congruence with our previous study^29^ which used data from the same two monkeys (but different hemispheres), and to avoid contamination from ‘slow gamma’^29^ which was prominent in Monkey 4. For the contrast study, gamma range was chosen between 20–75 Hz. This was done to accommodate peak frequency for all stimulus contrast values, since gamma peak frequency has been to shown to decrease considerably with a reduction in stimulus contrast^27^.

Power in gamma and high-gamma ranges were calculated by first averaging the power values obtained from the PSDs in the corresponding frequency ranges, excluding line noise (60 Hz for Monkeys 1, 2 and 50 Hz for Monkeys 3, 4) and their harmonics. Change in power for each stimulus condition was then calculated as follows:

2 $$\Delta Powe{r}_{i}=10\,({\log }_{10}S{T}_{i}-B{L}_{ave})$$

where ST_i_ is the power summed across the frequency range of interest for stimulus condition *i*, and BL_ave_ is the baseline power averaged across conditions $(B{L}_{ave}=average({{\log }}_{10}B{L}_{i}))$.

Preferred orientation and orientation selectivity for each recording site were calculated using the following equations:

3 $$Preferred\,orientation={{\tan }}^{-1}\left(\frac{{\Sigma }_{i=1}^{N}{R}_{i}\,{\sin }(2{\theta }_{i})}{{\Sigma }_{i=1}^{N}{R}_{i}\,{\cos }(2{\theta }_{i})}\right)$$

4 $$Orientation\,selectivity=\frac{|{\Sigma }_{i=1}^{N}{R}_{i}{e}^{(j\cdot 2{\theta }_{i})}|}{{\Sigma }_{i=1}^{N}{R}_{i}}$$

where ${\theta }_{i}$ and ${R}_{i}$ are the orientations and sum of the power in gamma band. N is the total number of orientation values (8).

The slopes (Fig. 4) were calculated for stimulus (200 to 400 ms for Monkeys 1, 2 and 250 to 500 ms for Monkeys 3, 4) and baseline (−200 to 0 ms for Monkeys 1, 2 and −500 to 0 ms for Monkeys 3 and 4) periods in high-gamma frequency range (150–250 Hz) by fitting the function $lo{g}_{10}(P)=m\ast lo{g}_{10}(f)+c$, where *P* is the PSD, f is the frequency, *c* is the constant or noise floor and *m* is the slope^41,62^. In this frequency range, the amplifier roll off is negligible, and therefore the slopes are similar with or without amplifier roll-off correction^41^. We also tested the amplifier noise floor by shorting the inputs and found the power to be at least an order of magnitude lower than the signal power. Therefore, the estimated slopes did not depend on the characteristics of the amplifier.

## Supplementary information

Supporting Information.

## Data Availability

The datasets analyzed during the current study are available from the corresponding author on reasonable request.
