# Supplementary material for: Comparison of tuning properties of gamma and high-gamma power in local field potential (LFP) versus electrocorticogram (ECoG) in visual cortex
Source: Sci Rep. 2020 Mar 25;10:5422. doi: 10.1038/s41598-020-61961-9 (PMC7096473; doi:10.1038/s41598-020-61961-9)
Supplement: Supplementary file 1 — Supporting Information. [file 41598_2020_61961_MOESM1_ESM.pdf]

Supporting Information

Title: Comparison of tuning properties of gamma and high-gamma power in local field potential (LFP) versus electrocorticogram (ECoG) in visual cortex

Running title: Comparison of gamma and hi-gamma tuning in LFP versus ECoG

Authors

Agrita Dubey[1,2] and Supratim Ray[1*]

Affiliations

[1]Centre for Neuroscience, Indian Institute of Science, Bangalore, India, 560012

Telephone +91 80 2293 3437, Facsimile +91 80 2360 3323

[2]Center for Neural Science, New York University, New York, USA, 10003

Corresponding author:

*Supratim Ray: sray@iisc.ac.in

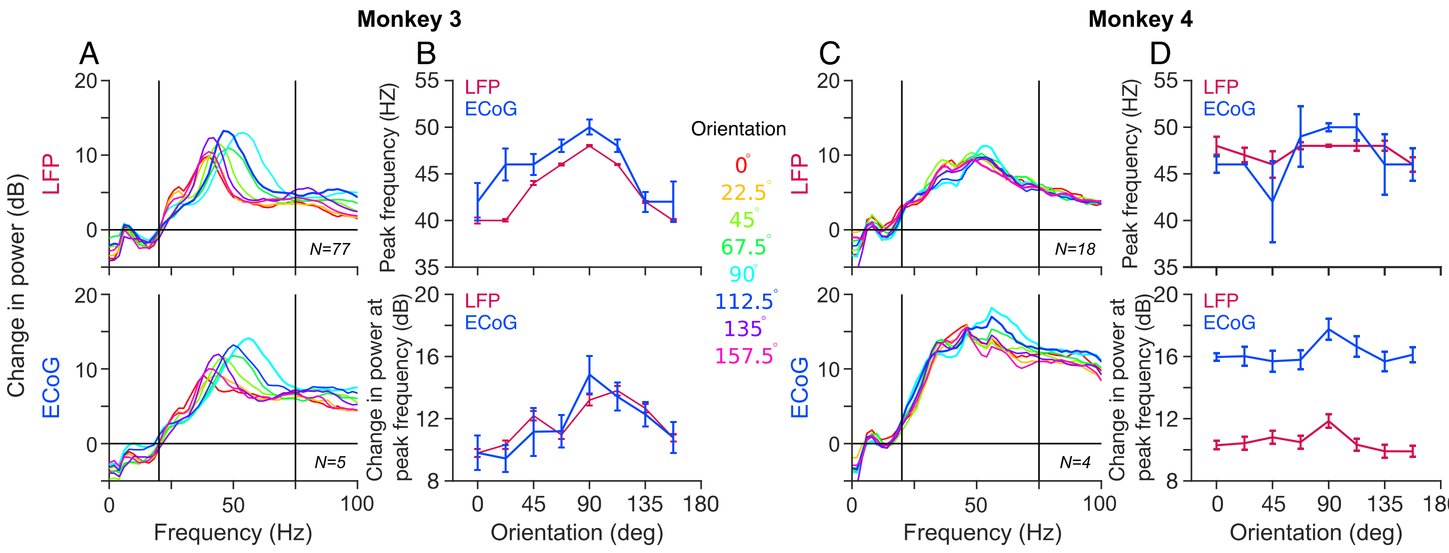

Supplementary Figure 1: Orientation tuning of LFP and ECoG in 20 – 75 Hz frequency range. **(A)** Average relative change in power spectra between 250 and 750 ms from baseline energy (-500 to 0 ms) for 77 LFP (top panel) and 5 ECoG recording sites (bottom panel) for Monkey 3. Eight colored traces are for eight different orientation values (labelled at the centre of Figure). **(B)** Average peak frequency in 20-75 Hz frequency range as a function of orientation (top panel) and mean change in power at peak frequency (bottom panel) across recording sites for LFP (magenta) and ECoG (blue). Error bar indicates SEs of the mean. **(C–D)** same as **A–B** but for 18 LFP and 4 ECoG recording sites in Monkey 4.

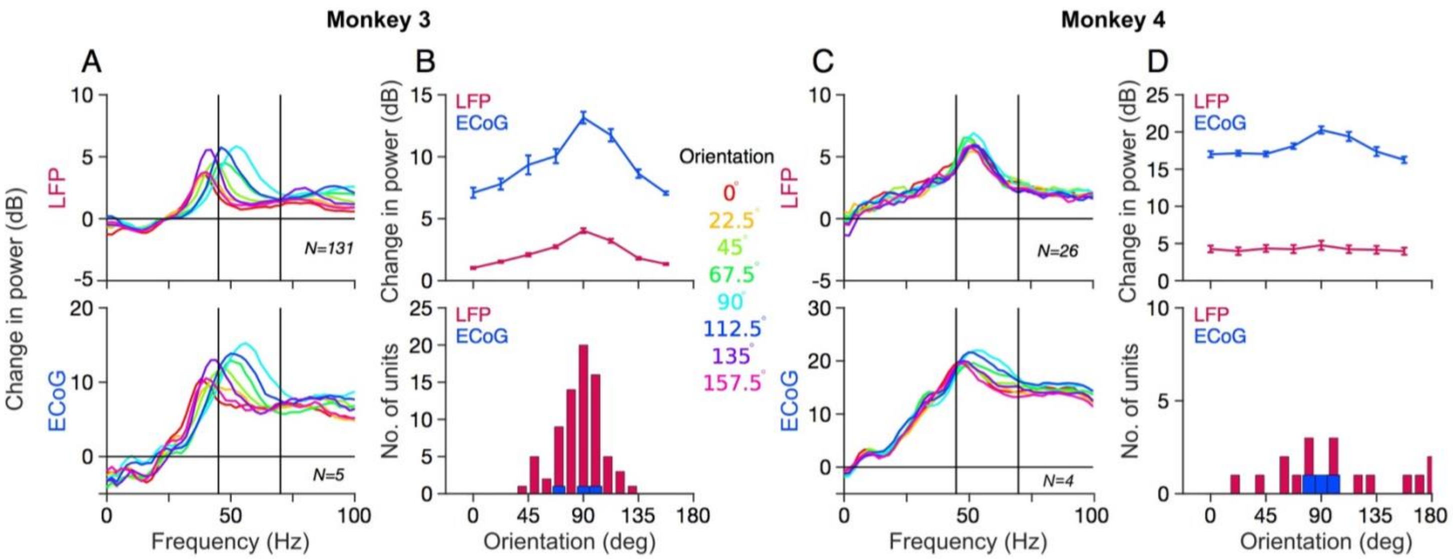

Supplementary Figure 2: Orientation tuning of gamma oscillations in LFP and ECoG after bipolar referencing. Same as Figure 5 but after bipolar referencing. The bipolar pairs were the adjacent pair of electrodes both in horizontal and vertical directions. The bipolar referenced signal was obtained by taking the difference between the pair. **(A)** Average relative change in power spectra between 250 and 750 ms from baseline energy (-500 to 0 ms) for 131 LFP (top panel) and 5 ECoG bipolar sites (bottom panel) for Monkey 3. Eight colored traces are for eight different orientation values (labelled at the centre of Figure). **(B)** Average change in gamma power as a function of orientation (top panel) and the histogram of orientation preference (bottom panel) across recording sites for LFP (magenta) and ECoG (blue). Error bar indicates SEs of the mean. **(C–D)** same as **A–B** but for 26 LFP and 4 ECoG bipolar sites in Monkey 4.
